# Guidance on Minimum Standards for Canine-Assisted Psychotherapy in Adolescent Mental Health: Delphi Expert Consensus on Health, Safety, and Canine Welfare

**DOI:** 10.3390/ani14050705

**Published:** 2024-02-23

**Authors:** Melanie G. Jones, Kate Filia, Simon M. Rice, Sue M. Cotton

**Affiliations:** 1 Centre for Youth Mental Health, University of Melbourne, Parkville, Melbourne, VIC 3052, Australia; kate.filia@orygen.org.au (K.F.); sue.cotton@orygen.org.au (S.M.C.); 2 Orygen, Parkville, Melbourne, VIC 3052, Australia; 3 Lead The Way Institute, Ferntree Gully, Melbourne, VIC 3156, Australia

**Keywords:** animal-assisted therapy, canine-assisted psychotherapy, adolescence, mental health, Delphi, standards, guidelines, health, safety, welfare

## Abstract

**Simple Summary:**

Including therapy dogs in adolescent mental health is increasingly popular; however, there is poor guidance to assist providers in developing high-quality interventions. This study recruited global experts in the field to agree on a set of minimum standards for health, safety, and welfare. A panel of 40 experts agreed that 32 items out of a possible 49 were important or essential to the minimum standards, including risk assessments, veterinary screening and preventative healthcare for dogs, and training in infection control and first aid for providers. Welfare measures included training providers to assess, document, and respond to dog (and human) wellbeing. Strict measures, including fecal testing, prohibiting raw food diets, and one-hour work schedules, were not included in the minimum standards, despite their common use in hospital/acute settings. Recommendations for providers are made.

**Abstract:**

As interest in animal-assisted therapy (AAT) and canine-assisted psychotherapy (CAP) grows, there are increasing calls for the management of related health, safety, and welfare concerns for canines, providers, and clients. Existing health and safety guidelines lack empirical support and are, at times, contradictory. Welfare is increasingly prioritized; however, tools to monitor and manage welfare are underutilized and under-reported. The aim of this study was to provide expert consensus on the minimum health, safety, and welfare standards required to develop and deliver a CAP group program to adolescents experiencing common mental health disorders. Diverse AAT experts were recruited globally. Using Delphi methodology, over two rounds, 40 panelists reached a consensus agreement to include 32 items from a possible 49 into the minimum standards. Health and safety measures included risk assessment, veterinary screening, preventative medicine, training in infection control, and first aid. Welfare measures included training in welfare assessment, documentation of welfare, and flexible, individualized responses to promote wellbeing. Intestinal screening for parasites and the prohibition of raw food were not supported. Flexible and individualized assessment and management of canine welfare were supported over fixed and time-limited work schedules. Clinical practice implications are discussed, and recommendations are made.

## 1. Introduction

Global interest in animal-assisted therapies (AAT) is increasing, as evidenced by the growing number of publications on the topic [1]. AAT is a goal-focused therapy, delivered and documented by a qualified professional within the scope of their professional practice, and assisted by the deliberate and meaningful inclusion of an animal [2]. A sub-set of AAT, canine-assisted psychotherapy (CAP), refers to mental health interventions that incorporate canines. Both AAT and CAP fall under the broader umbrella of animal-assisted interventions (AAIs), including both formal therapies and informal activities and visits [3].

With increased interest and participation in AAIs, the regulation of therapeutic and visiting animals is vital [4,5]. Concerns highlighted in the literature include a lack of standardized (universal) safety protocols [6,7,8], poor hygiene practices, and unregulated dogs entering facilities, e.g., [9]. These concerns extend to health, safety, and infection control in visiting dog organizations [10], schools [11], and healthcare settings [12,13]. Issues may arise from poor adherence to or understanding of best practice guidelines [11,13,14,15,16,17].

Zoonoses (infectious diseases that can pass between humans and animals) are one area of AAI requiring further research. Evidence suggests that canines working in AAI should be regularly treated for zoonoses, including internal and external parasites, but recommendations are conflicted on the regular assessment of intestinal pathogens, for example, via fecal screening [18,19,20]. Limited empirical data exist regarding the transmission of zoonotic illness between people, canines, and the environment in AAI [16,21,22]. Close contact between people and their pet dogs in home environments (e.g., kissing, bed-sharing) has been identified as one zoonotic risk factor [23]. The feeding of raw animal products is another risk factor, with dogs fed raw or home-cooked food being more likely to carry zoonotic intestinal pathogens [19,24,25,26]. Some researchers have suggested that pets’ fur, including cats and dogs, could serve as possible vectors for disease transmission [18,27,28]. However, limited research has explored human infection rates following AAI, with inconsistent findings [21]. Importantly, asymptomatic canines infected with concerning or treatment-resistant pathogens may work with clients [13,19,28], which is of particular concern in healthcare settings [8].

Vulnerable clients such as young children, older or pregnant people, and people with immune-compromisation may face an increased risk of illness following exposure to zoonoses and/or of suffering more severe illness [23], particularly if canines are permitted to lick [25,28]. Consequently, established guidelines in acute settings, such as the Society for Healthcare Epidemiology of America (SHAE) guidelines [20], typically recommend strict washing/grooming protocols for the canines, hand hygiene, reducing client exposure to canine saliva, and excluding the consumption of raw products of animal origin. Routine veterinary health screening, parasite prevention, vaccinations, and the exclusion of symptomatic dogs (or handlers) are also recommended [16,20,22,29].

Beyond infection control, there are limited empirical data on risks, such as fear, phobia, aversion, injuries, or allergies [5,21]. Using practice-based evidence, some researchers have made recommendations regarding risk assessment and safety in AAI [6,7]. Brelsford and colleagues published the Lincoln Education Assistance with Dogs (LEAD) risk, safety, and welfare assessment tools [6]. These templates were developed and trialed in primary/elementary school settings. Standardized risk assessment and management protocols may also be beneficial, assisting with the identification of risks, their likelihood and severity, and risk control options such as management or mitigation. Using a matrix, the level of risk is then identified, e.g., [30]. These are easily adapted to AAI in diverse settings.

Health and safety are closely linked to welfare, as deficiencies in one domain often lead to deficits in the other, e.g., [31,32,33,34,35,36,37,38]. Despite the international growth of AAI, gaps remain in understanding welfare, particularly animal welfare. Traditionally, animal welfare referred to the absence of distress and/or suffering, but current insights emphasize the rich emotional lives of animals [39,40]. Models of animal welfare now focus on positive emotional states, incorporating wellbeing and enrichment, and recognizing the importance of the environment and relationships. These include the ‘five domains’ model [41,42] and the ‘one health’ concept [43,44,45]. Consequently, the scientific study of canine welfare in human–animal interactions has grown to include not just the study of physiological parameters of stress but hormones and neurotransmitters reflective of positive emotion states [38,40].

Regular physiological assessments of therapy dogs are impractical in clinical practice, whereas behavioral ethograms, handler training, and supervision are more appropriate [34]. Adequate training in welfare is especially important given that dog owners and handlers may completely miss, minimize, or rationalize subtle stress signs in their dogs or show bias to inflate their dog’s capacity for and enjoyment of AAI [33,38,40,46,47,48]. Reassuringly, recent reviews of canine welfare in AAI have concluded that adequately trained and supervised dog-handler teams experience no significant welfare risks [34,35], particularly in predictable environments [49].

In this study, our focus is specifically on CAP delivered to adolescents in the form of group therapy. Our previous research indicates that CAP interventions may be particularly effective for this population group, providing novel and engaging interventions during a time when engagement may be challenging and when mental health interventions have the capacity to divert lifelong negative impacts [50]. There is limited information available describing how therapy dogs respond to groups of young people. Reviews by Ng [34] and Glenk and Foltin [35] report potential stress increases during interactions with a large number of children (e.g., in the playground), children under 12, and those with ADHD. Subtle signs of stress (lip licking, ears back) have been observed when therapy dogs interact with groups of young adults (college students) [51]. Yet, other studies have found that interactions with groups of diverse adults may increase stress in some dogs but not others. To our knowledge, no empirical studies have explored the specific health, safety, or welfare implications of delivering CAP to adolescents in a community (non-acute) setting.

Given the lack of available empirical data, in this study, we seek to establish expert consensus on minimum standards for developing and delivering a CAP group intervention for adolescents with regard to health, safety, and welfare.

## 2. Materials and Methods

**Figure 1:**
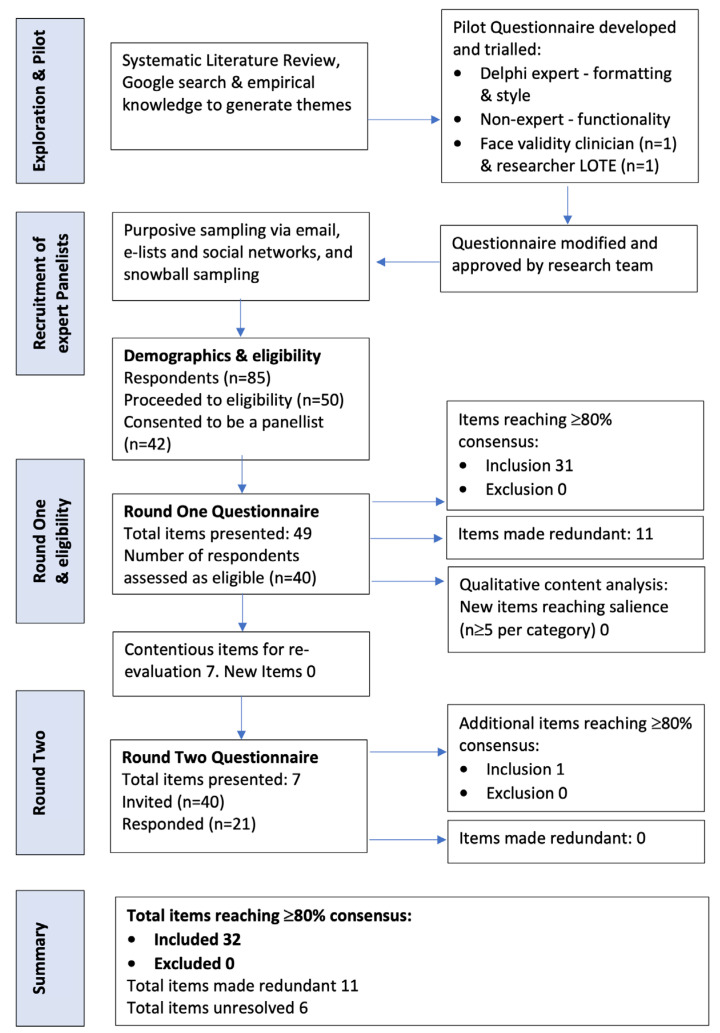
Delphi consensus on health, safety, and welfare in CAP.

This study forms one part of a larger research project examining intervention development and quality assurance in CAP [50]. The Delphi methodology, a well-established approach [52,53,54], was employed to obtain expert consensus through successive data collection rounds, addressing contentious areas identified in a systematic literature review [55]. Ethics approval for the research project was granted by the University of Melbourne Human Research and Ethics Committee (1853284), and guidance on Conducting and Reporting Delphi Studies (CREDES) was followed [56]. The methodology for the research project has been reported in detail elsewhere [50]. See Figure 1 for an overview of this study.

Panelists were recruited globally for their expertise in AAT and comprised academics and/or mental health clinicians from diverse locations, professional backgrounds, and species specializations. This ensured a diversity of opinion [52], a representative range of expertise across the AAT sector [50], and was likely to achieve a panel size sufficient for statistical stability [52]. Recruitment was conducted online via targeted methods (published authors, mailing lists, university departments) and a snowballing approach (social media, interest groups, AAT organizations). Demographic, consent, and eligibility information was collected. The eligibility criteria consisted of the following: (1) employment in an academic or teaching role with at least 5 years of experience in the study of AAT in mental health (or related field) with at least one peer-reviewed publication or thesis on AAT in mental health; and/or (2) qualifications and license or registration as a mental health professional with a minimum of 5 years of experience in the direct delivery of AAT.

A questionnaire was developed following initial validation and trialing to gather expert consensus on relevant topics over a maximum of three rounds (see Figure 1). Using an online platform (Qualtrics), AAT experts provided answers to items identified by the research team and made suggestions for further items to be evaluated in subsequent rounds. Expert panelists were asked to provide their opinions on *health and safety* and *welfare*. Specifically, panelists were asked, “*If you were developing a guideline for the minimum acceptable standards for a manualized CAP group intervention for adolescents experiencing common mental health disorders (including depression, anxiety and adjustment disorder) how would you rate the importance of the following*”. Free text fields were included in round one for additional suggestions to the researchers.

Each item presented was rated on a scale of (1) *irrelevant to the minimum standards* (and should therefore be excluded) to (5) *essential to the minimum standard* (and therefore should be included). A consensus agreement for inclusion was reached when 80% or more panelists rated the item as *important* (4) or *essential* (5). Exclusion was reached when 80% or more panelists rated the item as *unimportant* (4) or *irrelevant* (5). A high rate of consensus (80%) [57] was chosen by the research team to ensure that only strongly supported items were included or excluded from the minimum standards.

A broad range of suggestions, comments, and clarifications were provided by panelists in round one. Given the large volume of responses, additional suggestions were considered salient when five or more experts commented within the same domain, category, or topic (e.g., ‘allergies’, ‘veterinary screening’), the topic was relevant to CAP, and was not already covered elsewhere in the questionnaire. Using qualitative content analysis [58], new items were identified for presentation in subsequent rounds. In accordance with Delphi methodology, in subsequent rounds, expert panelists were provided with both an aggregate summary of the previous round’s results and a copy of their own responses [52]. This provided the opportunity for panelists to compare their responses with the aggregate. Expert panelists were also re-presented with any items remaining contentious from previous rounds and any new items for rating.

## 3. Results

### 3.1. Cohort Characteristics

A total of 40 eligible experts formed the panel from an initial pool of 85 respondents (see Figure 1). Panelists reported broad expertise in AAT, including clinical practice (77.5%) and/or academia (researcher 57.5%, teaching role 52.5%). The years of experience reported were commonly 5–10 years (45%); however, 30% reported having 16 or more years of experience. Panelists employed a range of species, primarily canines (77.5%), equines (37.5%), farm animals (15%), and felines (12.5%). Similar to other studies of AAI providers [14,33,59,60], participants in this study were more likely to be female (95%), Caucasian (95%), middle-aged (mean age 48.58), and residing in Western countries (USA 42.5%, Australia 30%, and Europe or UK 22.5%); see Table 1. Data collection was ceased after round two (21 panelists) due to attrition. There was insufficient statistical power to perform sub-group analyses of panelists.

### 3.2. Consensus Overview

Of the 49 items presented in this study, expert panelists reached a consensus agreement (≥80%) to include 32 items in the minimum standards across the domains of health and safety (see Table 2) and welfare (see Table 3). Rates of consensus were generally high, with 10 of the 32 items achieving 100% consensus agreement for inclusion. No items were excluded from the minimum standards. Six items did not reach a consensus agreement for inclusion or exclusion and remained contentious (see Supplemental Information Table S1). No suggestions from panelists reached inclusion (salience (*n* ≥ 5) plus novelty) in round one; therefore, no additional (new) items were presented to panelists in round two. In round two, one re-presented item reached a consensus agreement for inclusion.

### 3.3. Health and Safety

Panel members agreed that, to meet minimum standards, providers must be trained in zoonotic infections, including methods to reduce risks, human first aid, and canine first aid. Providers must also be up to date with recommended (human) vaccinations (relevant to the geographic area and population group) (see Table 2).

Regarding the canines working in the manualized intervention, expert panelists agreed that they must be up to date on recommended vaccinations by a licensed veterinarian, up to date on internal and external parasite control (e.g., fleas, ticks, worms), and have regular vet checks and clearance when working (every 6–12 months). The dogs must not work when obviously unwell (e.g., vomiting, diarrhea) or when obviously unhappy or displaying behavioral changes indicative of not wanting to engage in client work. Zoonotic clearance must be obtained from a vet prior to returning to work following illness. The exclusion of canines on antibiotics or antimicrobials even if otherwise appearing well (e.g., prophylactic treatments or to manage chronic/recurring conditions, e.g., itchy ears) did not reach a consensus (include 58.8%, exclude 41.4%). However, expert panelists agreed that canines with illness, injury, or disability must obtain veterinary clearance prior to working with clients. There was no consensus that only sterilized (de-sexed, neutered, spayed) canines can work with clients (include 29.4%, exclude 41.2%); however, expert panelists did reach a consensus agreement that canines must not work when on heat/in season.

To manage cross-contamination and allergies, panelists agreed that clients must use adequate hand hygiene before and after contact with the canine. This was in preference to using antiseptic wipes on canines between each client in the group (include 58.8%, exclude 17.7%) or between each group of clients (include 58.8%, exclude 29.4%). Expert panelists agreed that canines must be prevented from licking the client’s mouth or eyes. This was in preference to the prevention of all licking (include 48.1%, exclude 18.5%) or clients being prevented from coming into contact with canine saliva, paws, ears, or peri-anal region, as they are potential infection sources (i.e., not allowed to feed, touch toys from dog’s mouth, handle dog’s face/ears, ‘shake’ paws, etc.) (include 25%, exclude 46.4%). Expert panelists did not reach a consensus agreement to include or exclude regular screening for intestinal infections (e.g., salmonella) (e.g., every 60–90 days) (include 47.0%, exclude 41.2%) or prohibit canines from eating any raw animal product within 90 days prior to contact with clients (include 29.4%, exclude 41.4%).

Regarding grooming, expert panelists agreed that canines must be hydrobathed (shampoo down to the skin) when obviously soiled or malodorous, rather than being hydrobathed regularly (weekly to fortnightly) (include 60.7%, exclude 21.4%) or within 24 h prior to client contact (include 21.4%, exclude 39.3%). It was further agreed that canines must be groomed (brushed/wiped) every workday, as required.

To manage and mitigate risk, expert panelists agreed that risk assessments for both the venue/environment (e.g., hazards to humans or animals) and client–animal interaction (e.g., suitability, zoonoses, physical harm) should occur. Incident reports should be completed for all injuries to humans or animals (including physical injuries such as a scratch and psychological injuries such as fear) and for all ‘near miss’ incidents (e.g., a situation where a canine or client intended or threatened to cause harm). Incident reporting should include a review of the risk and future planning for risk management or mitigation. Panelists agreed that any human injury (bite, scratch) should be washed with soap and running water, followed by medical or first aid care and incident reporting, as required. There was greater support for injuries to be washed with soap and running water for 45 s (in accordance with hand hygiene advice (e.g., https://www.hha.org.au/hand-hygiene/what-is-hand-hygiene) (accessed 14 June 2023) over being washed for 5 min (in accordance with SHAE guidelines) [20], although both met consensus agreement for inclusion.

### 3.4. Welfare

Expert panelists universally agreed that providers of the manualized intervention must be trained in canine welfare and body language (see Table 3). In addition to any formal assessments, re-assessments, or ongoing training required for canine–human team certification, the expert panelists agreed that canine welfare must be assessed and maintained during clinical practice.

Universal support was given to welfare being informally assessed by provider observations during work. Also meeting consensus agreement was that canine welfare is regularly assessed and documented by provider observation (such as non-standardized questionnaires) and kept in the canine’s health record. This was in preference to the use of formal observational measures (e.g., video recording and subsequent expert coding of stress, behavioral questionnaire from the PAT-WAT) (include 53.6%, exclude 7.2%) and standardized welfare assessment tools (e.g., PAT-WAT, including behavioral coding and physiological testing) (include 60.7%, exclude 17.8%).

To maintain canine welfare, expert panelists agreed to a flexible response to the observed behavior/wellbeing of the individual canine and that canines should be free to engage, disengage, or rest (e.g., off-lead) during sessions. There was some support for canine welfare being maintained by adhering to strict guidelines, e.g., work duration of no more than one hour, rest breaks, and recovery days (irrespective of individual canine differences); however, this did not meet consensus (include 61.5%, exclude 11.5%). Suggestions from expert panelists were that welfare guidelines should be in place (*n* = 5); however, they must be responsive to the individual dog/situation (*n* = 5). These suggestions were already included in the questionnaire and did not result in new items being generated.

In order to adequately prepare the venue, canine, and client, expert panelists agreed that animal familiarization with the venue, setting, or location should occur prior to commencing client interactions (in preference to during group therapy; include 48.4%, exclude 35.5%). Client–animal familiarization, however, should occur during the initial phases of therapy and is seen as integral to the therapeutic process, as opposed to client–animal familiarization prior to commencing treatment (include 57.6%, exclude 33.3%). A consensus agreement was also reached that a suitability assessment of the venue/environment should occur (e.g., access, temperature, rest areas, the ability for animals to express natural behavior).

The expert panelists agreed that human and animal welfare should be given equal importance. This was in preference to human welfare (not safety) being given priority over animal welfare (include 7.1%, exclude 53.6%) and animal welfare (not safety) being given priority over human welfare (include 22.2%, exclude 40.7%).

## 4. Discussion

This study provides essential guidance to AAT providers on the safe integration of canines into psychotherapeutic treatments with adolescents. Given the scarcity of empirical evidence, we have provided expert consensus on minimum standards for health, safety, and canine welfare, the first of its kind outside of the hospital/acute sector. This guidance provides a foundational framework for providers to identify and implement minimum standards in clinical practice.

### 4.1. Key Findings

#### 4.1.1. Health and Safety

Existing health and safety guidelines for visiting dogs have been generated for the acute and hospital sectors based on expert consensus, most notably the SHAE guidelines developed by the Society for Healthcare Epidemiology of America [20]. With limited empirical evidence, however, it is unclear if such protocols are warranted for therapy dogs working in non-acute and community settings. Our study has further highlighted these issues, with experts agreeing that a range of infection control measures are warranted (as outlined in Table 2), including vaccinations, veterinary and zoonotic clearance, parasite control, hand hygiene, and exclusions for behavioral non-consent (not wanting to work), illness, and estrus. However, it is worth noting that consensus was not achieved regarding the prohibition of the consumption of raw animal products, despite this being recommended in most acute sector guidelines [8,16,20,22,29] and the LEAD risk assessment tools trialed in schools [6]. Additionally, routine screening for intestinal parasites, though widely employed by organizations coordinating visiting dogs [10], was not universally recommended in the SHAE guidelines [20] or supported by the experts in our study. The prohibition of all licking and the strict avoidance of canine saliva were not considered essential or important to the minimum standards, with experts agreeing only that canines should be prevented from licking clients’ mouths and eyes.

It is possible that these results indicate a lack of awareness of zoonotic risk factors; however, given the expertise of the panel, this seems unlikely. Alternatively, it may reflect the level of risk aversion of providers in clinical practice. This includes balancing ‘acceptable risk’ against client preference, whereby providers acknowledge that participants may already have close contact with animals in their day-to-day lives [25,61] and have the capacity to provide informed consent with regard to the risk of zoonoses. It may also reflect a deference by providers to their veterinarians. For example, the expert panelists did not reach a consensus to exclude canines taking antibiotics/antimicrobials if asymptomatic but did agree that canines with illness, injury, or disability obtain veterinary clearance and that zoonotic clearance is required following illness. This ensures that veterinarians can identify any concerning health or zoonotic issues for the individual canine prior to approving them for therapy work.

Alternatively, these results may reflect the practicalities of clinical practice, including the logistical constraints of strict measures such as daily bathing, expensive fecal testing every three months, the prohibition of licking, which anecdotally many participants solicit [25], and the prohibition of raw foods, which some argue have health benefits [26]. This is certainly an area for further research to develop a greater understanding of the risk profile of zoonoses in the non-acute or community sector and with ostensibly ‘healthy’ participants.

Also somewhat contentious is the frequency with which a canine should be hydrobathed (washed with shampoo down to the skin). Some acute sector guidelines support bathing within 24 h prior to client contact [10,29], whilst others recommend bathing only when required [20]. Experts in this study agreed that it was important or essential that canines were groomed (e.g., wiped down, brushed) every workday; however, complete hydrobathing was only required when the canine was obviously soiled or malodorous. This ensures that allergens and pathogens from the coat are kept to a minimum, whilst also maintaining the health of the canine’s skin and coat by preventing over-bathing.

Training and education of providers in human first aid and canine first aid met the criteria for inclusion in the minimum standards. It is generally recognized that ongoing education and qualification in these areas ensures that providers remain current with emerging best practices, with many jurisdictions providing clear guidelines for refresher training (e.g., annual re-training in human cardiopulmonary resuscitation). Standardized training in zoonoses and risk reduction are not as widely available but also met the inclusion criteria. It is likely, therefore, that this training would best be provided within an AAT-specific curriculum [50]. Experts also recognized the importance of handler health, agreeing that providers should be covered by relevant vaccinations.

Several risk assessment and incident reporting measures also met the criteria for inclusion in this study. These are broadly in line with established risk frameworks that seek to identify, mitigate, or manage risk, e.g., [6,30]. It is important to assess the environment and venue in which the CAP will occur, as well as the specific client–animal interactions themselves. Reviews of health, safety, and welfare regularly highlight the factors that should form part of these assessments, including, for example, temperature, access to water, areas to retreat and rest, allergy management, waste management, appropriate greetings/handling/interactions, the ability to express species-appropriate behavior, hygiene practices, cleaning protocols, and so on. Importantly, risk assessments should include respect for the individual canine’s consent to work or not to work [6,38,40]. Equally important is the evaluation of any adverse event or potential adverse event (‘near-miss’) in order to establish the antecedents of the event and identify mitigation or management strategies for the future [1,7]. This is supported by the incident reporting processes agreed upon by the expert panelists.

#### 4.1.2. Welfare

Understanding animal welfare is important from an ethological perspective and a clinical practice perspective [62]. That is, how is welfare operationalized? How is it understood (assessed) and implemented? What belief systems or biases do providers (or participants) hold that impact their adherence to policy or procedure? A number of factors should be considered and are supported by the current study, most notably the adequate training of providers in canine welfare and body language. This is perhaps the single most important method for enhancing canine welfare [34], allowing for individual variation, and helping to minimize handler bias [5,33,63]. Important too are the ongoing assessment and documentation of canine welfare so that providers are aware of and able to respond effectively to the therapy dog’s wellbeing over the short, medium, and long term. Models such as the integrative model of human–animal interactions (IMHAIs) [64] provide a framework for handlers to understand the emotional expression of their therapy dogs and how these are impacted by the environment and by their own emotional responses (via emotional contagion). In particular, increasing the dog’s sense of felt safety can reduce the likelihood of them becoming dysregulated, thereby reducing the likelihood of unsafe behavior.

Interestingly, expert panelists in this study agreed only that ‘informal observations’ made by providers and subsequently documented in the canine’s health record were essential to the minimum standard. Despite welfare assessment tools being available, no published CAP studies reported using a welfare assessment tool as part of a study or intervention methodology [55]. Nor was there support in the current study for the use of formalized or standardized measures of welfare, including the AAI-specific tool, the Pet Assisted Therapy Welfare Assessment Tool (PAT-WAT) [34,65]. This tool comprises a self-administered behavioral questionnaire, formal behavioral analysis and coding (from video footage), and physiological measures (cortisol). Despite their potential efficacy in a research setting, physiological measures are generally prohibitive in clinical practice, and neither physiological measures nor behavioral coding were supported by experts in the current study. In addition, the behavioral questions in the PAT-WAT elicit responses for obvious welfare challenges (e.g., refusal to follow commands, aggression). It is well-reported in the literature that therapy dogs typically show only subtle signs of stress (e.g., licking, looking away, avoidance), and reports of significant welfare challenges are largely absent [34,35,40,51,66]. No suggestions were made by experts in support of any other standardized tools cited in the literature [34,63,66], such as the Canine Behavioral Assessment and Research Questionnaire (C-BARQ) [67,68], validated quality of life (QOL) measures [69], or QOL scales modified specifically for therapy dog evaluation [70]. Together, these findings indicate that current welfare and behavior assessment tools are not widely utilized (or reported) in clinical trials and may not be useful in clinical practice.

To effectively maintain canine welfare and respond to any concerns, experts in this study advocate flexible responses to provider observations. This recognizes that individual dogs have varying preferences and need to express natural behavior. Despite advocacy from proponents as a method to manage welfare [6,10], fixed schedules of work (e.g., one-hour limits) are not supported by our study. While our results indicate that guidelines are important, these are best adapted to individuals in their contexts [70]. It is also important to balance safety with welfare considerations. For example, it has been recognized that being on a leash can be detrimental to therapy dog welfare [71]; however, many AAI organizations require dogs to be leashed at all times for safety [10,20,34].

Other strategies identified by our study as being important or essential to maintaining canine welfare include suitability assessments of and animal familiarization with the venue prior to engaging in client work. This has been highlighted in the literature as an important way to mitigate canine stress in AAI [34,35,37,66]. Equal importance should be given to the welfare of animals and humans in CAP. This balance ensures that neither clients nor dogs are exploited for the preference or benefit of the other.

### 4.2. Clinical Implications and Recommendations for Canine-Assisted Psychotherapy in Adolescent Mental Health

Providers delivering CAP interventions to adolescents should seek training in health and safety that includes both human and canine first aid qualifications. Comprehensive training in zoonoses, including methods to reduce risk, is also vital, given that effective zoonoses education can reduce infection risk [72]. Our data, however, do not support the level of infection control measures recommended in the acute sector. We therefore recommend that providers consider a graded or stepped approach to infection control whereby more strict measures are implemented as the vulnerability of participants increases. For example, the minimum standards identified in this study are implemented with healthy, non-vulnerable participants with the capacity to provide informed consent. This includes the provision of accessible and easy-to-understand information about risks and methods to reduce risk. Participants who are hygiene-compromised (by age or disability) are provided with increased support to comply with measures, for example, increased supervision and supported hand hygiene. When participants are considered vulnerable (e.g., those with compromised immunity), providers should consider implementing more strict measures, including, for example, the prohibition of raw animal product diets and/or licking or seeking specific informed consent. This ensures that providers are confident that participants genuinely understand the nature of the risks and that exposure to saliva from canines who consume raw animal products increases those risks.

A strong and enduring relationship with the canine’s veterinarian is also an important health, safety, and welfare measure for CAP providers. The canine’s veterinarian plays an important role in maintaining health, safety, and wellbeing, not only for canines but also in protecting participants from potential zoonotic illness. This relationship further acknowledges the importance of multidisciplinary input in AAT in line with One Health principles [25,43].

Providers should be supported in developing effective risk assessment tools that are updated when the work changes, such as a new environment or a new population group. These tools should be accompanied by incident reporting tools that are modified to include the unique dynamics involved in CAP, including the role of canines, providers/handlers, and participants. While generic risk and reporting tools are widely available in workplaces, AAT trainers and supervisors should support the identification and mitigation of specific risks associated with CAP.

Providers should also be supported in developing appropriate job descriptions and work schedules for their therapy dogs based on each individual dog’s welfare. This includes the need for canines to be ‘free to engage, disengage or rest (e.g., off-lead) in sessions’. Importantly, canines who are permitted to work off-lead with participants must be assessed for safety and suitability to work off-lead [63]. While visiting dog organizations typically stipulate one–two-hour work limits, e.g., [10], anecdotally, therapy dogs working in psychotherapeutic settings frequently work days of more than one to two hours and up to four or even more days in a week. In many instances, the ‘work’ day may not differ significantly from a non-workday, including periods of rest, play, and gentle interactions. What is of greater importance is the type of work, how much this work varies from the individual canine’s baseline behavioral preferences, and how much of the canine’s natural behavioral choices are impacted by the work [34,66,70]. For example, a working-breed dog who prefers active play may easily engage with a group of active adolescents for an hour multiple times per day without fatigue. A non-sporting or toy-breed dog who prefers gentle pats, however, may fatigue more readily and retire for the day after a single session.

Assessing an individual canine’s welfare, including subtle stress signals, using behavioral ethograms (detailed, individualized descriptions of behaviors) may have greater utility than existing welfare assessment tools [73,74]. They may also have greater utility in assessing positive welfare states. These include the canine’s willingness to be involved in the work (e.g., consent testing) [40,63], engagement and affiliative behaviors [34], and recognition of their own individual preferences, e.g., for high- or low-intensity interactions with different people, activities, and environments [34]. This knowledge should be used to develop canine care plans to enhance wellbeing both in-session and in-between sessions, e.g., [6,75]. Handler stress can also have a negative impact on canine stress [34,35]. Conversely, good attachment and a strong relationship between the handler and canine can confer benefits to both the animal and the intervention [17,38]. In particular, the development of attunement and empathy between the handler and animal (via the processes of emotional contagion and empathic responding) can improve both safety and animal welfare [64]. The handler–canine relationship must therefore form part of the overall risk assessment and wellbeing plan.

### 4.3. Strengths and Limitations

This study brings together global expertise to provide essential guidance to CAP providers working with adolescents experiencing common mental health disorders outside of the acute/hospital sector. We provide clearly defined standards in areas that have been historically marked by contention and ambiguity. The resulting guidance is a practical framework that is directly applicable to clinical practice and of significant utility to providers, trainers and supervisors, and governing/professional bodies.

However, it is essential to acknowledge the limitations of the study, in particular the substantial attrition of panelists between rounds one and two. This was largely attributed to the significant number of items presented to experts as part of this research project, which in its entirety comprised over 200 items per round. Nevertheless, it is notable that consensus for this study was rapidly achieved (within one round) for most items (32 out of a total of 49 items). Our data indicate that experts were readily able to identify those elements of health and safety that were important or essential for providers in clinical practice. There was also a significant female bias in the study, and a substantial proportion of respondents resided in the USA and identified as Caucasian. These findings are largely consistent with the demographic findings of other surveys of AAT providers, e.g., [14]. Nevertheless, it is possible that if the survey had been translated into languages other than English, the demographic distribution may have been more diverse. Finally, given the specificity of the research, that is, CAP groups for adolescents, it is not clear how these findings may translate to other population groups or settings.

## 5. Conclusions and Future Directions

Health, safety, and welfare are essential components of CAP practice. These encompass health screening and clearance, risk assessments, welfare assessments, and wellbeing plans for canines, providers, clients, and the environment in which the CAP occurs. Importantly, our data do not support the same degree of zoonotic risk mitigation as presented in existing acute and hospital sector guidelines. Similarly, our study found that welfare and wellbeing strategies must be individualized, and while guidelines must exist, fixed schedules of work were not supported. Provider training and education were highlighted as central to the effective identification and management of health, safety, and welfare in CAP.

It is important to acknowledge that the expert panelists in this study are unlikely to have had extensive formal training in infection control or epidemiology, and with a lack of empirical evidence, infection control in ‘healthy’ populations warrants further study. Future research should also explore intervention development, such that providers have guidance on the ‘how’, ‘where’, and ‘why’ of CAP for adolescents. It is only through the development of theoretically sound, replicable interventions that feasibility, acceptability, tolerability, and efficacy can be established.

## Figures and Tables

**Table 1:** Demographic details of expert panelists.

Demographics	Response	Frequency (*n*)	Percentage %
Country of residence	United States of America	17	42.5
	Australia	12	30
	Europe	8	22.5
	UK	1	2.5
	South America	1	2.5
	Undisclosed	1	2.5
Cultural identity/ethnicity	Caucasian (e.g., “White”, “Anglo”, “European”, “Australian”)	30	65
	Hispanic	1	2.5
	Ashkenazi Jewish	1	2.5
	Undisclosed	8	20
Language(s) spoken	English only	28	70
	English in addition to other language(s)	9	22.5
	Undisclosed	3	7.5
Gender identity	Female	37	92.5
	Male	1	2.5
	Non-binary	1	2.5
	Undisclosed	1	2.5
Age range	Youngest 27 years		Mean Age
	Eldest 76 years		48.58 years
**AAT Expertise**	**Response**	**Frequency (*n*)**	**Percentage %**
Primary occupation	Teacher/educator	12	52.5
	Researcher	13	60
	Provider of AAT	31	77.5
Primary species	Dog	31	77.5
	Horse	15	37.5
	Farm animal (“chicken”, “goat”, “donkey”, “sheep”)	7	17.5
	Cat	5	12.5
	Small animal (“rat”, “hamster”)	5	12.5
	Bird/aviary	2	5
	Reptile	2	5
	Other (“dolphin”)	1	2.5
Total years AAT experience	5–10 years	18	45
	11–15 years	10	25
	16 years or more	12	30
Mental health qualifications (e.g., “Psychology”, “Counselling & Psychotherapy”, “Social Work”, etc.)	Secondary/diploma	0	0
	Tertiary/degree	2	5
	Post-graduate	38	95
AAT training	Self-directed CPD/CE (e.g., conferences, books, workshops)	15	37.5
	Short course, certificate	25	62.5
	Certified, accredited, or registered with an organization	24	60
	Tertiary degree (or equivalent)	3	7.5
	Post-graduate (e.g., thesis)	6	15
	Supervised practice/internship	3	7.5
	Dog trainer, dog behavior training	5	12.5
	AAT consultant, supervisor, legislator, conference speaker, postgraduate course developer	8	20
AAT peer-reviewed publications	Nil	8	20
	1–4	18	45
	5–10	4	10
	11 or more	10	25

**Table 2:** Health and safety: items meeting consensus for inclusion into the minimum standards for the delivery of a CAP group intervention for adolescents experiencing common mental health disorders.

Item ^1^	Consensus ^2^	Mean and SD ^3^	Median Rating ^3^	Round
**Health and Safety**				
Providers trained in zoonoses and risk reduction	100%	4.7 (0.5)	5	1
Providers trained and qualified in human first aid	89.3%	4.4 (0.8)	5	1
Providers trained in canine first aid	96.4%	4.4 (0.6)	4	1
Providers up to date with all relevant (human) vaccines	85.8%	4.4 (1.0)	5	1
Canines up to date on all relevant vaccines	100%	4.9 (0.3)	5	1
Canines up to date on internal/external parasite control	100%	4.9 (0.3)	5	1
Canines obtain regular vet clearance (6–12 monthly)	96.4%	4.8 (0.5)	5	1
Canines do not work when showing signs of illness	100%	5.0 (0.2)	5	1
Canines do not work when unhappy/behavior indicating not wanting to work	100%	4.9 (0.3)	5	1
Zoonotic clearance to work obtained following illness	82.2%	4.5 (0.8)	5	1
Canines with illness, injury, or disability obtain vet clearance to work	87.5%	4.4 (0.8)	5	1
Canines do not work when on heat/in season	88.2%	4.4 (1.3)	5	2
Clients use hand hygiene before and after canine contact	92.8%	4.5 (0.9)	5	1
Canines are prevented from licking client’s mouth or eyes	81.5%	4.3 (0.9)	5	1
Canines are hydrobathed when obviously soiled/malodorous	85.7%	4.3 (1.1)	5	1
Canines are groomed every workday	92.8%	4.5 (0.7)	5	1
Risk assessment of venue/environment	100%	4.8 (0.5)	5	1
Risk assessment of client–animal interaction	97.0%	4.9 (0.3)	5	1
Incident reporting completed for ALL injuries (human/animal) (physical/psychological)	92.9%	4.6 (0.6)	5	1
Incident reporting completed for all ‘near miss’ incidents	92.9%	4.4 (0.6)	4.5	1
Incident reporting includes review of risk, plus future management, or mitigation	100%	4.9 (0.4)	5	1
Any minor injury washed (5 min) + first aid and reported	89.3%	4.4 (0.9)	5	1
Any minor injury washed (45 s) + first aid and reported	96.3%	4.7 (0.6)	5	1

^1^ Items here are abbreviated. ^2^ Inclusion criteria are ≥80% consensus agreement as *important* or *essential.* ^3^ M = mean, SD = standard deviation. Rating from 1 (unimportant) to 5 (essential) to the minimum standards.

**Table 3:** Welfare: items meeting consensus for inclusion into the minimum standards for the delivery of a CAP group intervention for adolescents experiencing common mental health disorders.

Item ^1^	Consensus ^2^	Mean and SD ^3^	Median Rating ^3^	Round
**Welfare**				
Providers are trained in canine welfare and body language	100%	4.8 (0.4)	5	1
Canine welfare is assessed by informal provider observations during work	100%	4.7 (0.5)	5	1
Canine welfare is assessed and documented by provider in canine health record	85.8%	4.2 (0.9)	4	1
Canine welfare is maintained by flexible response to observations	89.2%	4.4 (0.8)	5	1
Canines are free to engage, disengage, rest (e.g., off-lead) in sessions	96.3%	4.7 (0.7)	5	1
Suitability assessment of venue/environment	100%	4.8 (0.4)	5	1
Animal familiarization with venue/setting *prior* to group therapy	84.9%	4.2 (1.0)	4	1
Client–animal familiarization occurs *during* group therapy (therapeutic process)	90.7%	4.4 (0.8)	5	1
Human and animal welfare are given equal importance	96.4%	4.8 (0.5)	5	1

^1^ Items here are abbreviated. ^2^ Inclusion criteria are ≥80% consensus agreement as *important* or *essential.* ^3^ M = mean, SD = standard deviation. Rating from 1 (unimportant) to 5 (essential) to the minimum standards.

## Data Availability

The data in this study form part of a larger, ongoing research project and as such are not available for public use. Please contact the research team directly for further information regarding data.

## Supplementary Materials

The following supporting information can be downloaded at: https://www.mdpi.com/article/10.3390/ani14050705/s1, Supplemental Information Table S1.
