# Supplementary material for: Guidance on Minimum Standards for Canine-Assisted Psychotherapy in Adolescent Mental Health: Delphi Expert Consensus on Health, Safety, and Canine Welfare
Source: Animals (Basel). 2024 Feb 23;14(5):705. doi: 10.3390/ani14050705 (PMC10930943; doi:10.3390/ani14050705)
Supplement: Supplementary file 1 [file animals-14-00705-s001.zip › animals-2867286-supplementary.pdf]

Guidance on minimum standards for canine assisted psychotherapy in adolescent mental health:

Delphi expert consensus on health, safety, and canine welfare.

Table S1 – Supplementary Information

Health & Safety

R1 No.	Round One Item	%	Mean SD Median	R2 No.	Round Two Item (where relevant)	%	Mean SD Median	Accepted
D1.2a 1a	**Providers are trained in zoonotic infections, and methods to reduce risks**	E 0 **I 100** N 0	M=4.7 SD=0.5 Med=5.0					Yes
1b	**Providers are first aid trained and qualified**	E 3.6 **I 89.3** N 7.1	M=4.4 SD=0.8 Med=5					Yes
1c	**Providers are trained in canine first aid**	E 0 **I 96.4** N 3.6	M=4.4 SD=0.6 Med=4.0					Yes
1d	**Providers are up to date with recommended (human) vaccinations (relevant to the geographic area and population group)**	E 3.6 **I 85.8** N 10.7	M=4.4 SD=1.0 Med=5.0					Yes
1e	**Any *minor human injury* (bite, scratch) is washed with soap and running water for 5 minutes (SHAE guidelines), then Medical or first aid care and incident reporting as required**	E 3.6 **I 89.3** N 7.1	M=4.4 SD=0.9 Med=5.0					Yes
1f	**Any *minor human injury* (bite, scratch) is washed with soap and running water for 45 seconds (in accordance with hand hygiene advice) followed by Medical or first aid car and incident reporting as required**	E 0 **I 96.3** N 3.7	M=4.7 SD=0.6 Med=5.0					Yes
1g	**Incident reporting includes review of risk, and future planning for risk management or mitigation**	E 0 **I 100** N 0	M=4.9 SD=0.4 Med=5.0					Yes

key

% - Percentage to E (exclude) I (include) or N (neither)
M= mean score and SD= standard deviation, where 1 is irrelevant and 5 is essential
Suggestions – Salient categories where 5 or more experts (N=5) made suggestions in the same category, topic or domain
Accepted – accepted for inclusion in the minimum standards, having reached consensus of 80% or greater as important (4) or essential (5) (approach consensus 70-70%, some support 60 – 69%)
Exclusion – having reached consensus of 80% or greater as irrelevant (1) or unimportant (2) (approaching consensus 70-70%, some support 60 – 69%)

Guidance on minimum standards for canine assisted psychotherapy in adolescent mental health:

Delphi expert consensus on health, safety, and canine welfare.

Table S1 – Supplementary Information

R1 No.	Round One Item	%	Mean SD Median	R2 No.	Round Two Item (where relevant)	%	Mean SD Median	Accepted
1h	**Incident reports are completed for all injuries to humans or animals (e.g., physical injury – scratch; psychological injury – fear)**	E 0<br>**I 92.9**<br>N 7.1	M=4.6<br>SD=0.6<br>Med=5.0					Yes
1i	**Incident reports are completed for all 'near miss' incidents (e.g., a situation where a canine or client intended or threatened to cause harm)**	E 0<br>**I 92.9**<br>N 7.1	M=4.4<br>SD=0.6<br>Med=4.5					Yes
1j	**Canines are up to date on recommended vaccinations by a licensed veterinarian**	E 0<br>**I 100**<br>N 0	M=4.9<br>SD=0.3<br>Med=5.0					Yes
1k	**Canines are up to date on internal and external parasite control (e.g., flicks, ticks, worms)**	E 0<br>**I 100**<br>N 0	M=4.9<br>SD=0.3<br>Med=5.0					Yes
1l	**Canines require regular vet checks and clearance when working (every 6-12 months)**	E 0<br>**I 96.4**<br>N 3.6	M=4.8<br>SD=0.5<br>Med=5.0					Yes
1m	Canines are prohibited from eating any raw animal product within 90 days prior to contact with clients	E 10.7<br>I 46.5<br>N 42.9	M=3.6<br>SD=1.2<br>Med=3.0	1.1	Canines are prohibited from eating any raw animal product within 90 days prior to contact with clients	E 41.4<br>I 29.4<br>N 29.4	M=2.9<br>SD=1.5<br>Med=3.0	
1n	**Canines do not work when obviously unwell (e.g., vomiting, diarrhoea)**	E 0<br>**I 100**<br>N 0	M=5.0<br>SD=0.2<br>Med=5.0					Yes
1o	Canines do not work when on antibiotics or antimicrobials even if otherwise appearing well (e.g., prophylactic treatments, or to manage chronic/recurring conditions e.g., itchy ears)	E 14.8<br>I 55.5<br>N 29.6	M=3.7<br>SD=1.3<br>Med=4.0	1.2	Canines do not work when on antibiotics or antimicrobials even if otherwise appearing well (e.g., prophylactic treatments, or to manage chronic/recurring conditions e.g., itchy ears)	E 41.1<br>I 58.8<br>N 0	M=3.2<br>SD=4.0<br>Med=1.5	

<u>**key**</u>
% - Percentage to E (exclude) I (include) or N (neither)
M= mean score and SD= standard deviation, where 1 is irrelevant and 5 is essential
Suggestions – Salient categories where 5 or more experts (N=5) made suggestions in the same category, topic or domain
Accepted – accepted for inclusion in the minimum standards, having reached consensus of 80% or greater as important (4) or essential (5) (approach consensus 70-70%, some support 60 – 69%)
Exclusion – having reached consensus of 80% or greater as irrelevant (1) or unimportant (2) (approaching consensus 70-70%, some support 60 – 69%)

Guidance on minimum standards for canine assisted psychotherapy in adolescent mental health:

Delphi expert consensus on health, safety, and canine welfare.

Table S1 – Supplementary Information

R1 No.	Round One Item	%	Mean SD Median	R2 No.	Round Two Item (where relevant)	%	Mean SD Median	Accepted
1p	**Canines do not work when obviously unhappy or displaying behavioural changes indicative of not wanting to engage in client work**	E 0<br>**I 100**<br>N 0	M=4.9<br>SD=0.3<br>Med=5.0					Yes
1q	**Zoonotic clearance is obtained from a vet prior to return to work following illness**	E 0<br>**I 82.2**<br>N 17.9	M=4.5<br>SD=0.8<br>Med=5.0					Yes
1r	Canines are screened for intestinal infections (e.g., salmonella) regularly (e.g., every 60-90 days)	E 14.2<br>I 46.4<br>N 39.3	M=3.6<br>SD=1.2<br>Med=3.0	1.3	Canines are screened for intestinal infections (e.g., salmonella) regularly (e.g., every 60-90 days)	E 41.2<br>I 47.0<br>N 11.8	M=3.2<br>SD=1.5<br>Med=3.0	
1s	**Canines with illness, injury or disability must obtain veterinary clearance prior to working with clients**	E 3.6<br>**I 87.5**<br>N 10.7	M=4.4<br>SD=0.8<br>Med=5.0					Yes
1t	Only sterilised (de-sexed, neutered, spayed) canines can work with clients	E 46.5<br>I 17.8<br>N 35.7	M=2.5<br>SD=1.2<br>Med=3.0	1.4	Only sterilised (de-sexed, neutered, spayed) canines can work with clients	E 41.2<br>I 29.4<br>N 0	M=2.8<br>SD=1.5<br>Med=3.0	
1u	Canines must not work when on heat/in season	E 7.2<br>I 60.7<br>N 32.1	M=4.0<br>SD=1.1<br>Med=4.0	1.5	**Canines must not work when on heat/in season**	E 11.8<br>**I 88.2**<br>N 0	M=4.4<br>SD=1.3<br>Med=5.0	Yes
1v	Canines are hydrobathed (shampoo down to the skin) within 24 hours prior to client contact	E 39.3<br>I 21.4<br>N 39.3	M=2.8<br>SD=1.3<br>Med=3.0	*Item redundant*				
1w	Canines are hydrobathed regularly (weekly to fortnightly)	E 21.4	M=3.6	*Item redundant*				

key

% - Percentage to E (exclude) I (include) or N (neither)
M= mean score and SD= standard deviation, where 1 is irrelevant and 5 is essential
Suggestions – Salient categories where 5 or more experts (N=5) made suggestions in the same category, topic or domain
Accepted – accepted for inclusion in the minimum standards, having reached consensus of 80% or greater as important (4) or essential (5) (approach consensus 70-70%, some support 60 – 69%)
Exclusion – having reached consensus of 80% or greater as irrelevant (1) or unimportant (2) (approaching consensus 70-70%, some support 60 – 69%)

Table S1 – Supplementary Information

R1 No.	Round One Item	%	Mean SD Median	R2 No.	Round Two Item (where relevant)	%	Mean SD Median	Accepted
		I 60.7 N 17.9	SD=1.4 Med=4.0	*Some support*				
1x	**Canines are hydrobathed when obviously soiled or malodorous**	E 7.1 **I 85.7** N 7.1	M=4.3 SD=1.1 Med=5.0					Yes
1y	**Canines are groomed (brushed/wiped) every workday, as required**	E 3.6 **I 92.8** N 3.6	M=4.5 SD=0.7 Med=5.0					Yes
1z	**Clients use adequate hand hygiene before and after contact with the canine**	E 3.6 **I 92.8** N 3.6	M=4.5 SD=0.9 Med=5.0					Yes
1aa	Cross-contamination measures are taken between clients (e.g., use of antiseptic wipes) NOTE: Clients are working together in a group setting	E 3.6 I 75.0 N 21.4	M=4.2 SD=1.0 Med=5.0	1.6	Cross-contamination measures are taken between clients (e.g., use of antiseptic wipes) NOTE: clients are working together in a group setting	E 17.7 I 58.8 N 23.5	M=3.8 SD=1.5 Med=5.0	
1bb	Cross-contamination measures are taken between GROUPS of clients (e.g., use of antiseptic wipes) I.e. the dog is wiped down prior to working with a new group of clients	E 7.4 I 77.7 N 14.8	M=4.1 SD=1.1 Med=4.0	1.7	Cross-contamination measures should be taken between GROUPS of clients (e.g., use of antiseptic wipes). I.e. the dog is wiped down prior to working with a new group of clients	E 29.4 I 58.8 N 11.8	M=3.3 SD=1.7 Med=4.0	
1cc	**Canines are prevented from licking the client's mouth or eyes**	E 3.7 **I 81.5** N 14.8	M=4.3 SD=0.9 Med=5.0					Yes
1dd	Canines are prevented from licking clients at all	E 18.5 I 48.1 N 33.3	M=3.6 SD=1.2 Med=3.0	*Item redundant*				

key

% - Percentage to E (exclude) I (include) or N (neither)
M= mean score and SD= standard deviation, where 1 is irrelevant and 5 is essential
Suggestions – Salient categories where 5 or more experts (N=5) made suggestions in the same category, topic or domain
Accepted – accepted for inclusion in the minimum standards, having reached consensus of 80% or greater as important (4) or essential (5) (approach consensus 70-70%, some support 60 – 69%)
Exclusion – having reached consensus of 80% or greater as irrelevant (1) or unimportant (2) (approaching consensus 70-70%, some support 60 – 69%)

Guidance on minimum standards for canine assisted psychotherapy in adolescent mental health:

Delphi expert consensus on health, safety, and canine welfare.

Table S1 – Supplementary Information

R1 No.	Round One Item	%	Mean SD Median	R2 No.	Round Two Item (where relevant)	%	Mean SD Median	Accepted
1ee	Clients are prevented from coming into contact with canine saliva, paws, ears, peri-anal region as they are potential infection sources (i.e., not allowed to feed, touch toys from dog's mouth, handle dog's face/ears, 'shake' paws etc)	E 46.4<br>I 25.0<br>N 28.6	M=2.8<br>SD=1.4<br>Med=3.0	*Item redundant*				
D1<br>1d<br>1p	**Risk assessment of venue/environment (e.g., hazards to humans or animals)**	E 0<br>**I 100**<br>N 0	M=4.8<br>SD=0.5<br>Med=5.0					Yes
D1<br>1d<br>1q	**Risk assessment of client-animal interaction (e.g., suitability, zoonoses, physical harm)**	E 0<br>**I 97.0**<br>N 3.0	M=4.9<br>SD=0.3<br>Med=5.0					Yes
2	***<u>Other Health and Safety Suggestions</u>***<br>None met inclusion criteria of n=5 per category							

<u>**key**</u>
% - Percentage to E (exclude) I (include) or N (neither)
M= mean score and SD= standard deviation, where 1 is irrelevant and 5 is essential
Suggestions – Salient categories where 5 or more experts (N=5) made suggestions in the same category, topic or domain
Accepted – accepted for inclusion in the minimum standards, having reached consensus of 80% or greater as important (4) or essential (5) (approach consensus 70-70%, some support 60 – 69%)
Exclusion – having reached consensus of 80% or greater as irrelevant (1) or unimportant (2) (approaching consensus 70-70%, some support 60 – 69%)

Guidance on minimum standards for canine assisted psychotherapy in adolescent mental health:

Delphi expert consensus on health, safety, and canine welfare.

Table S1 – Supplementary Information

Welfare

R1 No.	Round One Item	%	Mean SD Median	R2 No.	Round Two Item (where relevant)	%	Mean SD Median	Accepted
D1.2b.1a	**Providers are trained in canine welfare and body language**	E 0<br>**I 100**<br>N 0	M=4.8<br>SD=0.4<br>Med=5.0					Yes
1b	**Welfare is informally assessed by provider observations during work**	E 0<br>**I 100**<br>N 0	M=4.7<br>SD=0.5<br>Med=5.0					Yes
1c	**Canine welfare is regularly *assessed* and documented by provider observation (such as non-standardised questionnaires) kept in the canine's health record**	E 3.6<br>**I 85.8**<br>N 10.7	M=4.2<br>SD=0.9<br>Med=4.0					Yes
1d	Welfare is regularly *assessed* with formal observational measures (e.g., video-recording and subsequent expert coding of stress; behavioural questionnaire from the PAT-WAT)	E 7.2<br>I 53.6<br>N 39.3	M=3.7<br>SD=1.0<br>Med=4.0		*Item redundant*			
1e	Canine welfare is regularly *assessed* and documented with standardised therapy dog welfare assessment tools (e.g., PAT-WAT) including behavioural coding and physiological testing	E 17.8<br>I 60.7<br>N 21.4	M=3.7<br>SD=1.2<br>Med=4.0		*Item redundant*<br>*Some support*			
1f	Canine welfare is *maintained* by adhering to strict guidelines, e.g., work duration of no more than one hour, rest breaks, recovery days (irrespective of individual canine differences).	E 11.5<br>I 61.5<br>N 26.9	M=3.8<br>SD=1.2<br>Med=4.0		*Item redundant*<br>*Some support*			
	<u>If yes, please describe:</u><br>Welfare guidelines should be in place (5) and must be responsive to the individual dog/situation (5)							

<u>key</u>

% - Percentage to E (exclude) I (include) or N (neither)
M= mean score and SD= standard deviation, where 1 is irrelevant and 5 is essential
Suggestions – Salient categories where 5 or more experts (N=5) made suggestions in the same category, topic or domain
Accepted – accepted for inclusion in the minimum standards, having reached consensus of 80% or greater as important (4) or essential (5) (approach consensus 70-70%, some support 60 – 69%)
Exclusion – having reached consensus of 80% or greater as irrelevant (1) or unimportant (2) (approaching consensus 70-70%, some support 60 – 69%)

Table S1 – Supplementary Information

1g	**Canine welfare is *maintained* by flexible response to observed behaviour/wellbeing of the individual canine**	E 3.6<br>**I 89.2**<br>N 7.1	M=4.4<br>SD=0.8<br>Med=5.0	Yes
1h	**Canines are free to engage or disengage, or to rest (e.g., off lead) during sessions**	E 3.7<br>**I 96.3**<br>N 0	M=4.7<br>SD=0.7<br>Med=5.0	Yes
1i	Human welfare (not safety) is given priority over animal welfare	E 53.6<br>I 7.1<br>N 39.3	M=2.2<br>SD=1.0<br>Med=2.0	*Item redundant*
1j	Animal welfare (not safety) is given priority over human welfare	E 40.7<br>I 22.2<br>N 37.0	M=2.6<br>SD=1.2<br>Med=3.0	*Item redundant*
1k	**Human and animal welfare are given equal importance**	E 0<br>**I 96.4**<br>N 3.6	M=4.8<br>SD=0.5<br>Med=5.0	Yes
D1<br>1d<br>1k	Clients and animals meeting under supervision to familiarise themselves *prior* to commencing therapy	E 33.3<br>I 57.6<br>N 9.1	M=3.3<br>SD=1.3<br>Med=4.0	*Item redundant*
D1<br>1d<br>1l	**Client and animal familiarisation occurs *during* the initial phases of therapy, and is seen as integral to the therapeutic process**	E 3.1<br>**I 90.7**<br>N 6.1	M=4.4<br>SD=0.8<br>Med=5.0	Yes
D1<br>1d<br>1m	**Animal familiarisation with the venue, setting or location, *prior* to commencing client interactions**	E 9.1<br>**I 84.9**<br>N 6.1	M=4.2<br>SD=1.0<br>Med=4.0	Yes
D1<br>1d<br>1n	Animal familiarisation with venue, setting or location can occur *simultaneously* with group therapy	E 35.5<br>I 48.4<br>N 16.1	M=3.0<br>SD=1.5<br>Med=3.0	*Item redundant*

key

% - Percentage to E (exclude) I (include) or N (neither)
M= mean score and SD= standard deviation, where 1 is irrelevant and 5 is essential
Suggestions – Salient categories where 5 or more experts (N=5) made suggestions in the same category, topic or domain
Accepted – accepted for inclusion in the minimum standards, having reached consensus of 80% or greater as important (4) or essential (5) (approach consensus 70-70%, some support 60 – 69%)
Exclusion – having reached consensus of 80% or greater as irrelevant (1) or unimportant (2) (approaching consensus 70-70%, some support 60 – 69%)

Table S1 – Supplementary Information

D1<br>1d<br>1o	**Suitability assessment of venue/environment (e.g., access, temperature, rest areas, ability for animals to express natural behaviour)**	E 0<br>**I 100**<br>N 0	M=4.8<br>SD=0.4<br>Med=5.0	Yes
2	***Other Suggestions for Welfare***<br>Nil met inclusion criteria of n=5 per category			

key

% - Percentage to E (exclude) I (include) or N (neither)
M= mean score and SD= standard deviation, where 1 is irrelevant and 5 is essential
Suggestions – Salient categories where 5 or more experts (N=5) made suggestions in the same category, topic or domain
Accepted – accepted for inclusion in the minimum standards, having reached consensus of 80% or greater as important (4) or essential (5) (approach consensus 70-70%, some support 60 – 69%)
Exclusion – having reached consensus of 80% or greater as irrelevant (1) or unimportant (2) (approaching consensus 70-70%, some support 60 – 69%)
